# Effect of spironolactone on vascular stiffness in hemodialysis patients: a randomized crossover trial

**DOI:** 10.48101/ujms.v127.8594

**Published:** 2022-05-23

**Authors:** Michael Eklund, Olof Hellberg, Hans Furuland, Yang Cao, Kent Wall, Erik Nilsson

**Affiliations:** a Department of Internal Medicine, Faculty of Medicine and Health, Örebro University, Örebro, Sweden; b Department of Medical Sciences, Uppsala University Hospital, Uppsala, Sweden; c Clinical Epidemiology and Biostatistics, School of Medical Sciences, Örebro University, Örebro, Sweden; d Department of Clinical Physiology, Faculty of Medicine and Health, Örebro University, Örebro, Sweden; e Department of Medical Epidemiology and Biostatistics, Karolinska Institutet, Stockholm, Sweden; f Faculty of Medicine and Health, Örebro University, Örebro, Sweden

**Keywords:** Mineralocorticoid receptor antagonists, pulse wave velocity, systolic function, diastolic function

## Abstract

**Background:**

The role of spironolactone treatment in hemodialysis patients is debated, but a survival benefit is suggested. Mineralocorticoids and chronic kidney disease have been linked to cardiovascular fibrosis. Therefore, we hypothesized that spironolactone would affect vascular stiffness, cardiac systolic, and diastolic function in hemodialysis patients.

**Methods:**

This was a randomized crossover study in hemodialysis patients supplemented with an echocardiographic case series. All outcomes reported here were secondary in the trial and were assessed without blinding. Block randomization and allocation determined treatment order. Participants received 50 mg spironolactone daily for 12 weeks and untreated observation for another 12 weeks. Pulse wave velocity (PWV) was measured before and after treatment and observation. Doppler-echocardiography was conducted before and after treatment. Systemic arterial compliance indexed to body surface area (SACi), left ventricular ejection fraction (LVEF), the peak early diastolic mitral inflow velocity (E), the peak late diastolic mitral inflow velocity (A), and the peak early diastolic myocardial lengthening velocity (E’) were measured. E/A and E/E’ were then calculated. Statistical analyses were conducted per protocol. A generalized linear mixed model with random participant effects was used for PWV. The Wilcoxon signed-rank test was used for echocardiographic variables.

**Results:**

Thirty participants were recruited, 18 completed follow-up, and 17 were included in PWV-analyses. Spironolactone treatment showed a tendency toward an increase in PWV of 1.34 (95% confidence interval: −0.11 to 2.78) m/s, which was not statistically significant (*P* = 0.07). There were no significant changes in any of the other variables (LVEF, E/A, E/Eʹ, or SACi).

**Conclusions:**

We found no evidence supporting an effect of 12-week administration of spironolactone 50 mg daily on vascular stiffness, cardiac systolic, or diastolic function in hemodialysis patients.

## Introduction

In recent years, there have been trials suggesting that mineralocorticoid receptor antagonists, such as spironolactone, reduce the risk of cardiovascular events in patients with end-stage renal disease (ESRD) (1, 2) and chronic kidney disease (CKD) with type 2 diabetes (3). However, the role of spironolactone in ESRD remains debated (4). Considering the high incidence of cardiovascular events in ESRD (5) and the possibility of spironolactone reducing this risk (4), it is of great interest to elucidate potential mechanisms of spironolactone in this high-risk population.

Fibrosis of the cardiovascular system has been associated with mineralocorticoids (6) and the presence of CKD (7). Animal studies suggest that mineralocorticoids promote the development of vascular fibrosis and stiffness (6), and an increase in vascular stiffness has a high predictability of cardiovascular events and mortality in ESRD (8). Mineralocorticoid levels also correlate with left ventricular mass (9, 10) and the risk of developing congestive heart failure in CKD (11). Furthermore, animal studies in rats have shown that the rate of cardiac fibrosis, as seen after myocardial infarctions, is increased in the presence of renal failure (7).

Mineralocorticoid receptor antagonists reduce vascular stiffness in various populations (9, 12, 13). However, the effect of spironolactone on the vascular system in patients undergoing hemodialysis is not well known. To our knowledge, only two studies have been published on this topic, suggesting that spironolactone has a beneficial effect on the vascular system in hemodialysis, as indicated by a reduction in the carotid intima-media thickness (14) and on markers of vascular calcification (15). In early-stage CKD, treatment with mineralocorticoid receptor antagonists reduces the risk of developing left ventricular hypertrophy (16) and reduces left ventricular mass (9). The results of studies evaluating the effects of spironolactone on left ventricular ejection fraction (LVEF) and left ventricular mass in ESRD are conflicting (2, 16–20).

We hypothesized that spironolactone would affect vascular stiffness in hemodialysis patients and aimed to describe potential effects on cardiac systolic and diastolic function.

## Material and methods

### Clinical measurements

This was a randomized crossover trial supplemented with a descriptive case series of Doppler-echocardiography variables. All variables reported here were secondary outcomes of the trial and were collected without blinding. Study design and sample size determination have been described in more detail elsewhere (21).

Participants were recruited from the dialysis units of Örebro University Hospital, Sweden, and Uppsala University Hospital, Sweden, between February 2013 and December 2014. Inclusion criteria were ≥ 18 years of age, hemodialysis sessions ≥ 3 times per week, compliance ≥ 3 months, and adequate cognitive function. Exclusion criteria were cardiac pacemaker, atrial fibrillation (persistent or permanent), plasma potassium > 6.5 mmol/L at any time during the last 2 months, life expectancy < 12 months, pregnancy, or breastfeeding.

Randomization was conducted in blocks of six using Clinstat (Statistical software, Martin Bland, United Kingdom) by a person not involved in the trial. The allocation was concealed by placing randomization results in opaque envelopes that were consecutively numbered and sealed, only to be revealed on the first visit following inclusion.

Participants were followed for two periods of 12 weeks separated by a 6-week washout phase. Randomization determined if participants started with treatment (oral spironolactone 50 mg daily) or untreated observation.

All study visits were scheduled before dialysis treatment on the day of the mid-week dialysis sessions. A total of four visits were planned, before and after both 12-week periods. At the first visit, comorbidities and concomitant medications were recorded from patient history, medical records, and the Swedish Renal Registry (https://www.medscinet.net/snr/). Laboratory samples were collected, blood pressure was assessed, and pulse wave velocity (PWV) was measured at all visits. Laboratory samples included high-density lipoprotein, low-density lipoprotein, and hemoglobin A1C and were analyzed by the accredited local hospital laboratory. Doppler-echocardiography was conducted before and after the treatment period only.

PWV, the gold standard in central vascular stiffness estimation (22), was measured using the SphygmoCor System (AtCor Medical Technical Group, Australia) and calculated using the SphygmoCor Software (SphygmoCor CvMS V9, AtCor Medical Group, Australia) algorithm. Measurements of PWV were repeated in triplicate, and the median value of these three measurements was used in statistical analyses.

Doppler-echocardiography was conducted by a single operator per study center using GE Vivid 9 with a 3.5 MHz probe (GE Vingmed Ultrasound A/S, Horten, Norway). Data were analyzed *en bloc* by a single evaluator using EchoPAC PC (version 112, GE Vingmed Ultrasound A/S, Norway).

LVEF was determined using the biplane Simpson’s method, and was used as a marker for systolic function (23). The E/Eˊ-ratio and E/A-ratio were used to assess diastolic function (24). E, the peak early diastolic mitral inflow velocity (cm/s), and A, the peak late diastolic mitral inflow velocity (cm/s), were determined using Doppler. Eˊ, the peak early diastolic myocardial lengthening velocity (cm/s), was measured in the lateral aspect of the left ventricle using tissue Doppler. Systemic arterial compliance indexed to body surface area (SACi) (mL/m^2^/mmHg), an indicator of vascular stiffness, was determined as described by Mohty et al. from stroke volume, body surface area, and brachial pulse pressure (25).

### Statistical analysis

Data were presented as median and interquartile range (IQR) for continuous variables and number and percentage for categorical variables. PWV and blood pressure data were analyzed using a generalized linear mixed model with fixed effects for treatment, intervention order and baseline measurements, as well as with random effects for participants (26). We considered PWV-data normally distributed, based on how it has been managed in previous studies in CKD (9, 12) and by visual inspection of the plotted data and regression model residuals. The Wilcoxon signed-rank test was used to assess statistical significance for change in Doppler-echocardiographic variables during treatment. Analyses were conducted per-protocol with list-wise deletion of incomplete cases in accordance with the study protocol, motivated by the objective of identifying mechanisms of action of spironolactone rather than a clinically significant treatment effect. All analyses were conducted using R-statistics v 3.6.1 (R Development Core Team, Austria), and a two-sided *P*-value < 0.05 was considered statistically significant.

### Ethical considerations

This study received ethical approval from *The Regional Ethical Review Board in Uppsala*, Sweden (reference number 2011/316), and was approved by The Swedish Medical Products Agency. It was registered at *The EU Clinical Trials Register* before the study started (http://www.clinicaltrialsregister.eu/; ID-number: 2011-002773-39). The Declaration of Helsinki was adhered to, and Good Clinical Practice was applied.

## Results

**Figure 1:**
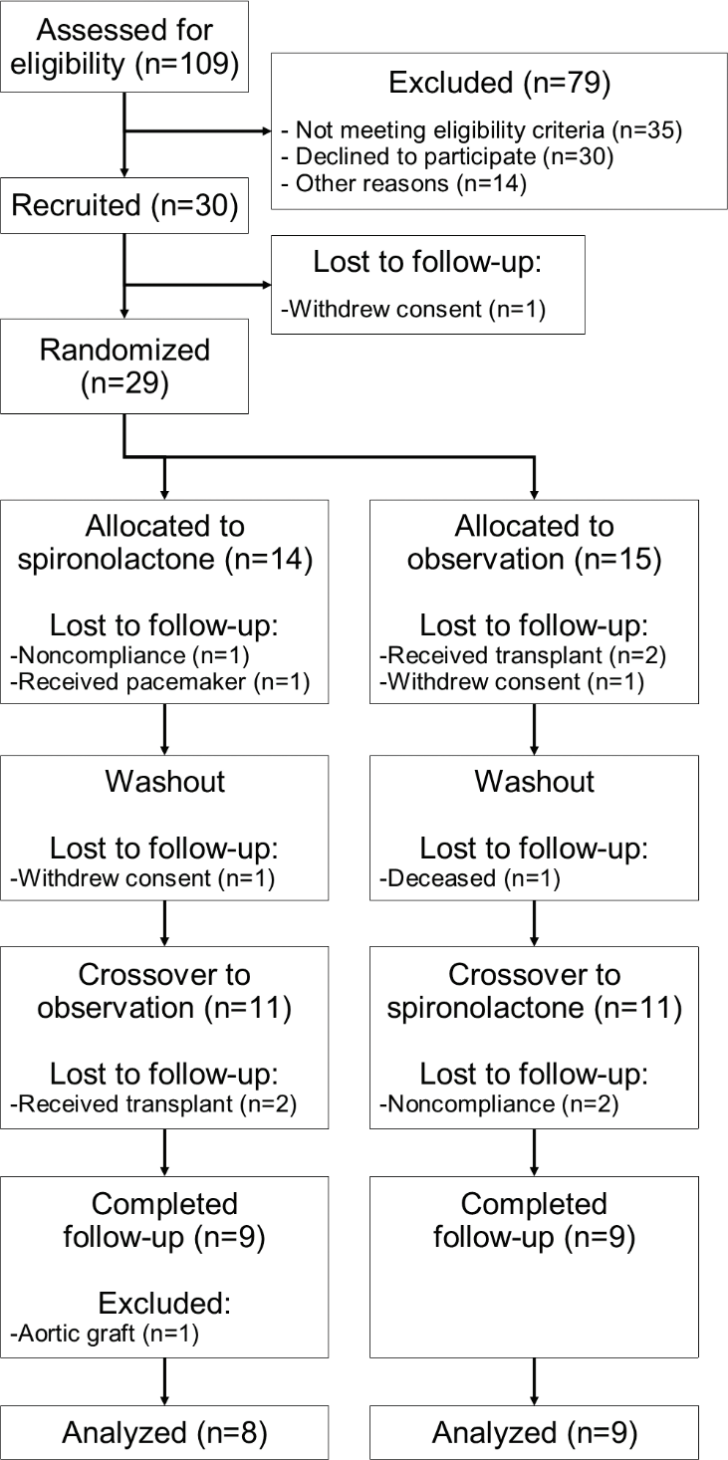
Study design, recruitment of participants, and follow-up for pulse wave velocity measurements.

A total of 30 participants were recruited, and 29 underwent randomization, of which 18 (60%) completed follow-up (Figure 1). The main reasons for being lost to follow-up were receiving a renal transplant (four cases), noncompliance (three cases), and withdrawal of consent (three cases). In the analysis of PWV, one further participant was excluded due to unreliable measurements as a consequence of an aortic graft, ending with 17 (57%) of all participants being included in the PWV analysis. Regarding the analysis of Doppler-echocardiographic variables, where only data before and after intervention were used, a total of 21 participants (70%) were included.

The majority of participants who conducted at least one study visit were male, 22 (76%), and had a median age of 66 (IQR: 62–70) years (Table 1). Participants who were lost to follow-up after the first visit were similar to complete cases regarding median age (67 vs. 66 years) and gender distribution (73% vs. 78% male) but had a longer median dialysis time (1,007 vs. 477 days).

**Table 1:** Baseline characteristics based on all 29 participants who underwent randomization.

Variable			Variable		
**Demographics**	**Number**	**%**	**Drug treatment**	**Number**	**%**
Male	22	76	RAS-inhibitors	17	59
Daily tobacco consumers	2	7	Betablockers	17	59
			Lipid lowering drugs	15	52
**Demographics**	**Median**	**IQR**	Loop diuretics	13	45
Age, years	66	62–70	Thiazide diuretics	1	3
Dialysis vintage, days	572	365–1,370	Vascular selective calcium channel blockers	9	31
**Comorbidities**	**Number**	**%**	**Laboratory analyses**	**Median**	**IQR**
Dyslipidemia	18	62	HDL, mmol/L	1.0	0.8–1.2
*Diabetes mellitus*	12	41	LDL, mmol/L	1.9	1.6–2.2
Diabetes type 1	2	7	HbA1C, mmol/mol	38	33–56
Diabetes type 2	10	34			
Myocardial infarction	6	21	**Blood pressure**	**Median**	**IQR**
Angina pectoris	8	28	Systolic, mmHg	140	120–165
Congestive heart failure	8	28	Diastolic, mmHg	74	68–90
Arrhythmic disorder	6	21			
Cerebrovascular event	4	14			
Peripheral vascular disease	4	14			

HbA1c: hemoglobin A1c; HDL: high-density lipoprotein; IQR: interquartile range; LDL: low-density lipoprotein; RAS: renin-angiotensin system.

**Figure 2:**
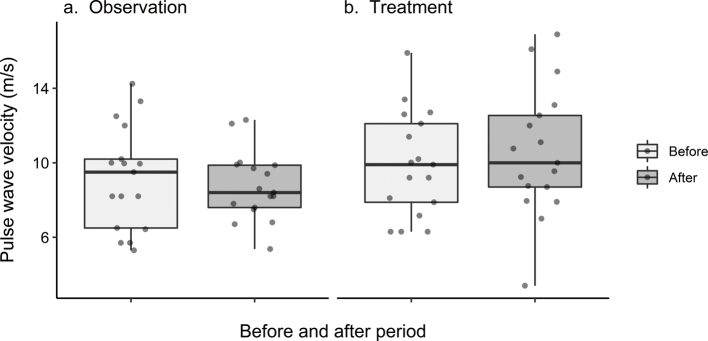
Absolute values of pulse wave velocity. Descriptive presentation of the distribution of the 17 observations of pulse wave velocity measurements (m/s) during different time points. Measurements are presented before (light grey) and after (dark grey) the 12-week study periods. Untreated observation is visualized in plot (a) and spironolactone treatment in plot (b). The horizontal lines represent medians, boxes the interquartile range, and whiskers the minimum and maximum values. Dots represent individual measurements.

**Figure 3:**
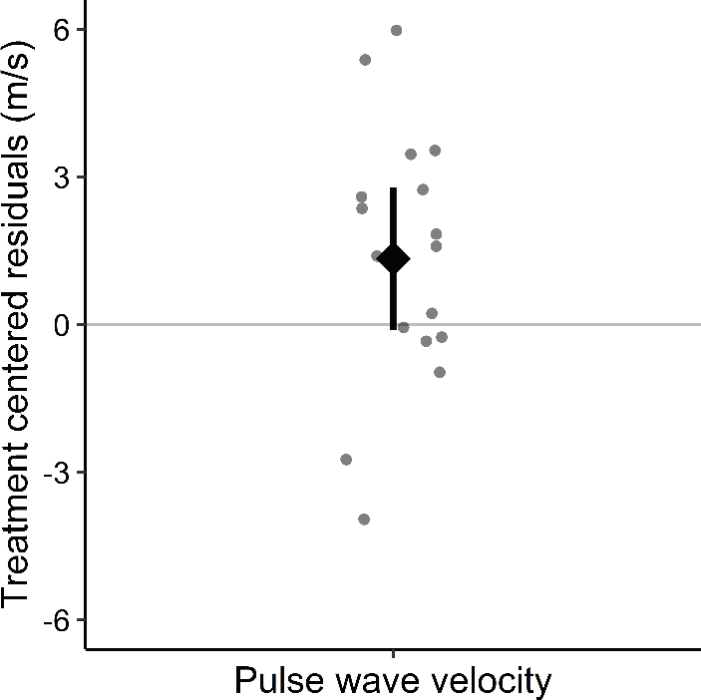
Effect of spironolactone on pulse wave velocity. Treatment-centered residuals for pulse wave velocity (m/s) based on 17 observations. The diamond signifies mean treatment effect (1.34 m/s), and horizontal bars represent the 95% confidence interval (−0.11 to 2.78 m/s). Dots indicate the individual treatment-centered residuals. The treatment-centered residuals represent the generalized linear mixed model with random effects residuals added to the treatment effect, which eliminates the model predicted random influences from the individual data points. Treatment, intervention order, and the baseline pulse wave velocity measurement were considered fixed effects, while the individual participants were considered random effects.

PWV remained stable over time, with a median change in PWV during the treatment period of 0.4 (IQR: −0.3 to 1.6) m/s and 0.0 (IQR: −1.6 to 1.1) m/s during observation (Figure 2). There was a slight, but not statistically significant, increase in PWV associated with spironolactone treatment, 1.34 (95% confidence interval [CI]: −0.11 to 2.78) m/s, *P* = 0.07 (Figure 3).

A post-hoc power analysis with PWV as the outcome using 17 subjects and the standard deviation of the change during treatment (1.94 m/s) yielded a minimal detectable difference of 1.4 m/s at 80% power.

Blood pressure did not change during treatment compared to observation; systolic blood pressure by 10 (95% CI: −2.9 to 23) mmHg, *P* = 0.12, and diastolic blood pressure by 4.2 (95% CI: −2.2 to 11) mmHg, *P* = 0.18.

**Figure 4:**
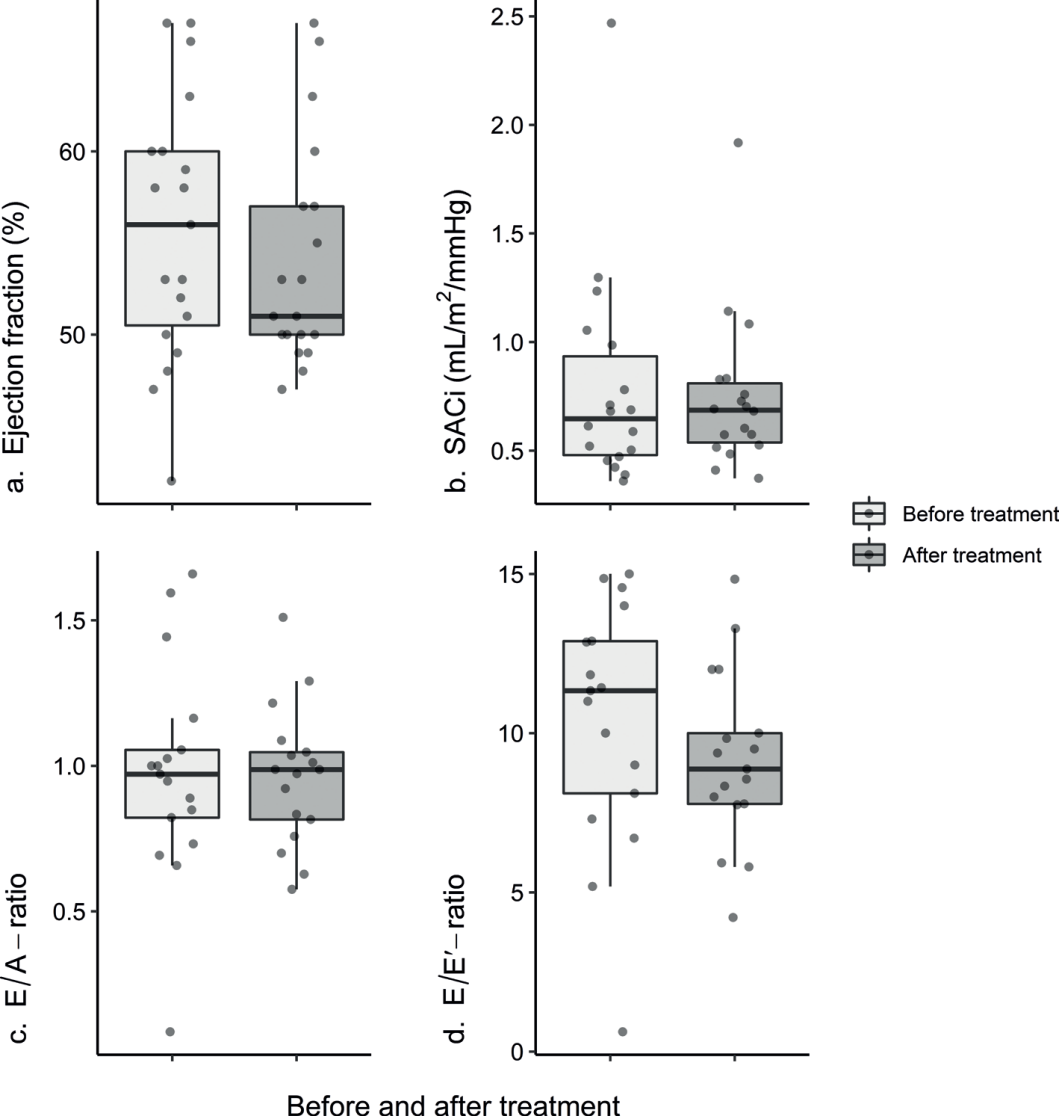
Effect of spironolactone on echocardiographic variables. Presenting case series data for measurements before (light grey) and after (dark grey) spironolactone treatment. Panel (a) presents measurements of ejection fraction for 19 participants (median change 0 [IQR: −7 to 3]%, *P* = 0.37). Panel (b) describes SACi values based on 18 participants (median change −0.01 [IQR: −0.14 to 0.09] mL/m^2^/mmHg, *P* = 0.61). Panels (c and d) depict measurements of diastolic function in 17 participants. The median change in E/A-ratio was −0.08 (IQR: −0.15 to 0.09), *P* = 0.38, and for E/Eʹ-ratio, it was −1.43 (IQR: −2.57 to 0.83), *P* = 0.06. Medians are represented by horizontal lines, interquartile range by boxes, and the minimum and maximum values by whiskers (not including outliers defined as being outside the 25th–75th percentile by more than 1.5 times the IQR). Dots represent individual measurements. All *P*-values were calculated using a two-sided Wilcoxon signed-rank test. IQR: interquartile range; SACi: systemic arterial compliance indexed to body surface area.

Doppler-echocardiographic variables remained stable during treatment and did not show any statistically significant change (Figure 4). However, a trend toward decreasing E/Eˊ-ratio was seen, with a median change of −1.43 (IQR: −2.57 to 0.83), but this did not reach statistical significance, *P* = 0.07.

## Discussion

This randomized crossover trial was designed to evaluate the effects of spironolactone on the cardiovascular system in hemodialysis patients. Using data from this study and a supplemental case series, we did not find any evidence supporting an effect of spironolactone 50 mg daily for 12 weeks on vascular stiffness, systolic cardiac function, or diastolic cardiac function in the participants.

To our knowledge, we are the first to report data on the effect of spironolactone on PWV, the gold standard in assessing vascular stiffness, in hemodialysis. Neither PWV nor SACi, another marker of vascular stiffness, showed any statistically significant change during treatment. These findings contrast previous findings in ESRD (14, 15) as well as findings of mineralocorticoid receptor antagonists in non-ESRD (9, 12). Vukusich et al. treated non-diabetic hemodialysis patients with spironolactone 50 mg three times per week (14). The treated population had a mean age of 60.1 years and a mean time in ESRD of 8.5 years. After 2 years of treatment, they described a reduced carotid intima-media thickness. Hammer et al. used spironolactone 50 mg daily or placebo for 40 weeks in patients on chronic hemodialysis to evaluate effects on markers of vascular calcification (15, 19). Their results only reached statistical significance in sensitivity analyses, indicating a possible beneficial effect of spironolactone on vascular calcification (15). The participants that underwent treatment had a mean age of 60.6 years, a median dialysis vintage of 35 months, and one-third had diabetes mellitus (19). In the study by Edwards et al., patients free from cardiovascular disease and diabetes with CKD stage 2–3 were treated with spironolactone 25 mg daily (9). The population had a mean age of 54 years in the treatment arm. Reductions in PWV and other markers of vascular stiffness were evident after 40 weeks of treatment. Finally, Boesby et al. used eplerenone in patients with CKD stage 3–4 and a mean age of 58 years, where approximately every fourth participant had diabetes (12). After 24 weeks of treatment, PWV was unchanged, but the pulse wave reflection was attenuated.

Differences in study design and included populations might explain why our results do not support previous findings. The potential vascular effects of spironolactone might depend on a longer duration of treatment than was the case in this study. Alternatively, the effect might have been mitigated by vascular pathological processes associated with diabetes mellitus, which was highly prevalent in our study, via non-mineralocorticoid pathways, such as advanced glycation end-products (27). Additionally, we used a higher dose of spironolactone than both of the aforementioned studies on vascular pathology that showed positive effects of treatment (9, 14) as well as the larger studies on mortality in hemodialysis (1, 2). Hammer et al. used the same dose used in the current trial and did not find any statistically significant effect on markers on vascular calcification in the primary analysis, only in sensitivity analyses (15). These differences in dosing and outcome might suggest that spironolactone has a nonlinear dose-response relationship to vascular pathology in hemodialysis. It is unlikely that extensive arterial ageing due to high age and extensive dialysis vintage at baseline could explain the lack of effect of spironolactone on vascular stiffness in this study. Our study population was only slightly older (median age 66 years) and had a relatively short dialysis vintage (572 days) compared to the previous trials (9, 12, 14, 15, 19).

In the descriptive analysis of LVEF and markers of diastolic function, we did not see any change during treatment. These findings are in line with recent findings on LVEF (18, 19, 28) and markers of diastolic function (18, 28) in ESRD. In contrast, other studies have shown an effect of spironolactone on LVEF in this population (2, 16, 17, 20). Edwards et al. further showed a beneficial effect of spironolactone on diastolic cardiac function in early-stage CKD (29). A possible explanation for the differences in results may be the frequency of renin–angiotensin system inhibitor use, which was > 80% in all studies showing an improvement in systolic or diastolic function (2, 16, 17, 20, 29). In comparison, it was < 60% in our study and in other studies that did not show any effect of spironolactone on these markers (18, 19, 28). This is supported by an animal study indicating that treatment with spironolactone in combination with the angiotensin-receptor blocker candesartan reduces cardiac remodeling to a more extensive degree than when monotherapy of the respective substances is applied (30). Therefore, we suggest that dual inhibition of the renin–angiotensin system and aldosterone might be needed for effects on left ventricular function to be evident.

Several limitations of this study must be acknowledged. First, although the number of subjects analyzed was sufficient based on the power analysis for long-term electrocardiographic changes, the sample size was relatively small, and many subjects were lost to follow-up resulting in relatively low power of detecting changes in the variables reported here. Second, the intervention period was relatively short. Nevertheless, studies of spironolactone in congestive heart failure have shown a reduced mortality and rehospitalization rate after 30 days from treatment start (31), suggesting that an effect on surrogate markers should have been possible to detect after 12 weeks of spironolactone treatment. Third, placebo treatment was not used due to high production cost, and blinding was not practiced for the variables reported here. Therefore, there is a possibility that the expectations of the participants and assessors could affect measurements. However, such an effect would likely be in the direction of improvement in the measured parameters. Finally, Doppler-echocardiographic measurements were only available before and after treatment, making the findings only hypothesis-generating.

Strengths of this study include its crossover design that provides confounding control per design and requires a smaller number of participants to attain the same power as in parallel-group studies. Also, all measurements were undertaken before the midweek dialysis session, which promotes a similar hydration status between visits, increasing the reliability of measurements and minimizing the risk of confounding.

In conclusion, our results did not support any effect of 12 weeks of spironolactone treatment on vascular stiffness, cardiac systolic, or diastolic function in hemodialysis patients. Future studies should consider the possibility of a nonlinear dose-response relationship for spironolactone treatment on vascular stiffness in hemodialysis patients.
